# In situ vaccination at a peripheral tumor site augments response against melanoma brain metastases

**DOI:** 10.1136/jitc-2020-000809

**Published:** 2020-07-19

**Authors:** Paul A Clark, Raghava N Sriramaneni, Won Jong Jin, Justin C Jagodinsky, Amber M Bates, Abigail A Jaquish, Bryce R Anderson, Trang Le, Jonathan A Lubin, Ishan Chakravarty, Ian S Arthur, Clinton M Heinze, Emily I Guy, Jasdeep Kler, Kelsey A Klar, Peter M Carlson, Kyung Mann Kim, John S Kuo, Zachary S Morris

**Affiliations:** 1 Department of Human Oncology, University of Wisconsin-Madison School of Medicine and Public Health, Madison, Wisconsin, USA; 2 Department of Neurological Surgery, University of Wisconsin-Madison School of Medicine and Public Health, Madison, Wisconsin, USA; 3 Department of Biostatistics and Medical Informatics, University of Wisconsin-Madison School of Medicine and Public Health, Madison, Wisconsin, USA; 4 Department of Neurosurgery Dell Medical School and Mulva Clinic for the Neurosciences, University of Texas at Austin, Austin, Texas, USA

**Keywords:** brain neoplasms, central nervous system neoplasms, immunotherapy, melanoma, radioimmunotherapy

## Abstract

**Background:**

Immune checkpoint inhibition (ICI) alone is not efficacious for a large number of patients with melanoma brain metastases. We previously established an in situ vaccination (ISV) regimen combining radiation and immunocytokine to enhance response to ICIs. Here, we tested whether ISV inhibits the development of brain metastases in a murine melanoma model.

**Methods:**

B78 (GD2^+^) melanoma ‘primary’ tumors were engrafted on the right flank of C57BL/6 mice. After 3–4 weeks, primary tumors were treated with ISV (radiation (12 Gy, day 1), α-GD2 immunocytokine (hu14.18-IL2, days 6–10)) and ICI (α-CTLA-4, days 3, 6, 9). Complete response (CR) was defined as no residual tumor observed at treatment day 90. Mice with CR were tested for immune memory by re-engraftment with B78 in the left flank and then the brain. To test ISV efficacy against metastases, tumors were also engrafted in the left flank and brain of previously untreated mice. Tumors were analyzed by quantitative reverse transcription-PCR, immunohistochemistry, flow cytometry and multiplex cytokine assay.

**Results:**

ISV+α-CTLA-4 resulted in immune memory and rejection of B78 engraftment in the brain in 11 of 12 mice. When B78 was engrafted in brain prior to treatment, ISV+α-CTLA-4 increased survival compared with ICI alone. ISV+α-CTLA-4 eradicated left flank tumors but did not elicit CR at brain sites when tumor cells were engrafted in brain prior to ISV. ISV+α-CTLA-4 increased CD8^+^ and CD4^+^ T cells in flank and brain tumors compared with untreated mice. Among ISV + α-CTLA-4 treated mice, left flank tumors showed increased CD8^+^ infiltration and CD8^+^:FOXP3^+^ ratio compared with brain tumors. Flank and brain tumors showed minimal differences in expression of immune checkpoint receptors/ligands or *Mhc-1*. Cytokine productions were similar in left flank and brain tumors in untreated mice. Following ISV+α-CTLA-4, production of immune-stimulatory cytokines was greater in left flank compared with brain tumor grafts.

**Conclusion:**

ISV augmented response to ICIs in murine melanoma at brain and extracranial tumor sites. Although baseline tumor-immune microenvironments were similar at brain and extracranial tumor sites, response to ISV+α-CTLA-4 was divergent with reduced infiltration and activation of immune cells in brain tumors. Additional therapies may be needed for effective antitumor immune response against melanoma brain metastases.

## Background

Brain metastases occur in 60% of patients with advanced melanoma and are clinically challenging because of their negative impact on quality of life and survival and because of the risks and costs of brain-directed treatments.^1^ In about half of patients, immune checkpoint inhibition (ICI) with α-programmed cell death protein-1 (PD-1) and/or α-cytotoxic T-lymphocyte-associated protein-4 (CTLA-4) can elicit a response against small, asymptomatic melanoma brain metastases,^1–3^ but many patients do not respond and require surgical resection and/or high-dose radiotherapy to treat melanoma brain metastases with additional treatment risks. These risks may be higher in patients taking ICIs^4^ and some patients undergoing treatment of melanoma brain metastases may require transient cessation of systemic therapies including ICIs. We are evaluating strategies to augment the antitumor immune response to ICIs using in situ vaccine (ISV) approaches.^5^ Here, we test whether these approaches may enhance antitumor immune response against melanoma brain metastases.

ISV is a therapeutic strategy to increase tumor-specific antigen presentation in a patient’s own tumor with the goal of stimulating and diversifying a powerful antitumor T cell response. By modulating immune tolerance and functional immunogenicity of tumor cells, focal radiation may serve as a method of ISV.^6–8^ In rare cases, radiation alone may result in ‘abscopal’ responses at distant non-irradiated tumor sites in patients with multiple metastases due to antitumor immunity stimulated by the ISV effect of radiation.^9^ Preclinical studies demonstrate that the ISV effect of radiotherapy may be leveraged to more consistently augment the local and systemic antitumor response by combination with immunotherapies such as ICIs.^7 8^ Clinical studies confirm that radiotherapy elicits ISV in cancer patients when combined with ICIs.^6^ Retrospective studies suggest safety for combinations of radiation and ICIs^10^ but prospective studies have not yet demonstrated a survival benefit or improved systemic tumor response with the addition of radiotherapy to ICIs.^11 12^ To increase the effectiveness of ISV approaches in priming antitumor immunity, we are testing treatment strategies that combine radiation and local delivery of immunotherapies in the irradiated tumor microenvironment.^5^

Tumor-specific monoclonal antibodies (mAbs) are a common and effective class of cancer therapeutics. Although often designed to target and block the function of specific cancer cell membrane proteins, mAbs are also capable of initiating an immune response by engaging Fc-ʏ receptors on natural killer (NK) cells to elicit cell-mediated tumor destruction and on myeloid or dendritic cells to activate mAb-facilitated antigen presentation.^13–19^ Immunocytokines (ICs) consist of a tumor-specific mAb fused to an immune stimulatory cytokine. ICs exert potent antitumor effects by targeting to tumors and locally stimulating the immune cells to selectively destroy cancer cells. The hu14.18–IL2 IC consists of IL2 fused to the hu14.18 mAb, which targets disialoganglioside D2 (GD2) expressed on the plasma membrane of some melanoma cells.^20^ In syngeneic mice bearing B78 melanoma tumors, which express GD2, we previously reported that combined treatment with radiotherapy (RT) and intratumor (IT) injection of IC markedly enhanced response compared with RT alone, IC alone, RT +IT-hu14.18 mAb or RT +IV hu14.18–IL2 IC.^5^ Following this combined treatment with RT and IT-IC, we observed complete resolution of single tumors in 71% of animals. This induced an in situ vaccination (ISV) effect resulting in a memory T cell response that rendered these animals resistant to further implantation with tumor cells from the originally rejected tumor, including parental B16 melanoma tumor cells lacking the initially targeted GD2 antigen. IT-IC +RT+α-CTLA-4 demonstrated significant benefit in survival and metastases in our previous work for B78 extracranial melanoma, as compared against monotherapy for each treatment as well as dual combinations.^5 21^ These studies demonstrated an ability to enhance the ISV effect of radiation by combining RT with IT-IC, and the capacity of this enhanced ISV to augment response to ICI.

Although combinatorial immunotherapeutic approaches are effective against extracranial metastatic disease and against brain metastases in some patients with cancer,^1 3 22^ there remains a paucity of data about how to most effectively use these approaches to eliminate brain metastases. Intracranial metastases introduce more therapeutic challenges due to unique central nervous system (CNS) immune organization^23^ and presence of the neurovascular blood–brain barrier, which limits penetration of chemotherapeutics and regulates systemic immune cell entrance.^24^ In this study, we investigated the efficacy of our RT +IT IC ISV regimen against a murine model of melanoma brain metastases.

## Methods

### Cell lines

B78-D14 (B78) melanoma was derived from B16 melanoma, as previously described^25^ and was obtained from Ralph Reisfeld (Scripps Research Institute) in 2002. B16-F10 melanoma was obtained from American Type Culture Collection (ATCC) in 2005. All cancer cells were cultured in Roswell Park Memorial Institute (RPMI)-1640 medium supplemented with 10% fetal bovine serum (FBS), 2 mmol/L L-glutamine, 100 U/mL penicillin and 100 µg/mL streptomycin, as previously described.^5^ Cell authentication was performed per ATCC guidelines using morphology, growth curves and Mycoplasma testing within 6 months of use and routinely thereafter.

### Murine tumor models

All animal studies were approved by the Institutional Animal Care and Use Committee at the University of Wisconsin – Madison. Female mice (C57BL/6) were purchased at 6–8 weeks of age from Taconic and used for all experiments. Mouse experiments were repeated in two or more independent trials with at least four animals per treatment group in each trial; aggregate number of animals (n) is indicated. C57BL/6-Tg (Foxp3 DTR/EGFP) 23.2 Spar/Mmjax ‘DEREG’ mice were purchased from the Jackson Laboratory (MMRRC stock #32050). Treg depletion with diphteria toxin was achieved following prior methodology^21 26^ by daily intraperitoneal (IP) injection of 1 µg diphteria toxin (Sigma) diluted in phosphate buffered saline (PBS) for 2 days at day 14 and 15 post-RT, to ensure presence of melanoma brain tumor prior to Treg depletion. At euthanasia, mice were dissected and grossly examined for brain tumor to verify no confounding effect of Treg depletion on autoimmunity and subsequent mortality.^27^

IC and therapeutic antibodies hu14.18-IL2 IC was provided by Apeiron Biologics.^28^ The hu14.18 antibody component of this IC targets GD2, which is expressed in many human melanomas.^29^ The B78 melanoma line is GD2^+^, whereas the parental B16 melanoma line is GD2^-^. α-CTLA-4 (clone 9D9) was provided by Bristol-Myers Squibb.

For depletion studies, IP injections of 500 µg α-CD4 (clone GK1.5, ATCC) + 500 µg α-CD8 (clone 2.43, ATCC) mAb was given at days −1, 3 and 7 relative to intracranial injection of tumor cells; 1 mg of rat IgG was administered as control.

### Radiation

Radiation was delivered to in vivo tumors using an X-RAD 320 (Precision X-Ray), as previously described.^5^ Mice were immobilized using custom lead jigs that exposed the dorsal right flank. For in vivo experiments, a single fraction 12 Gy dose of radiation was delivered.

### In situ vaccination

**Figure 1:**
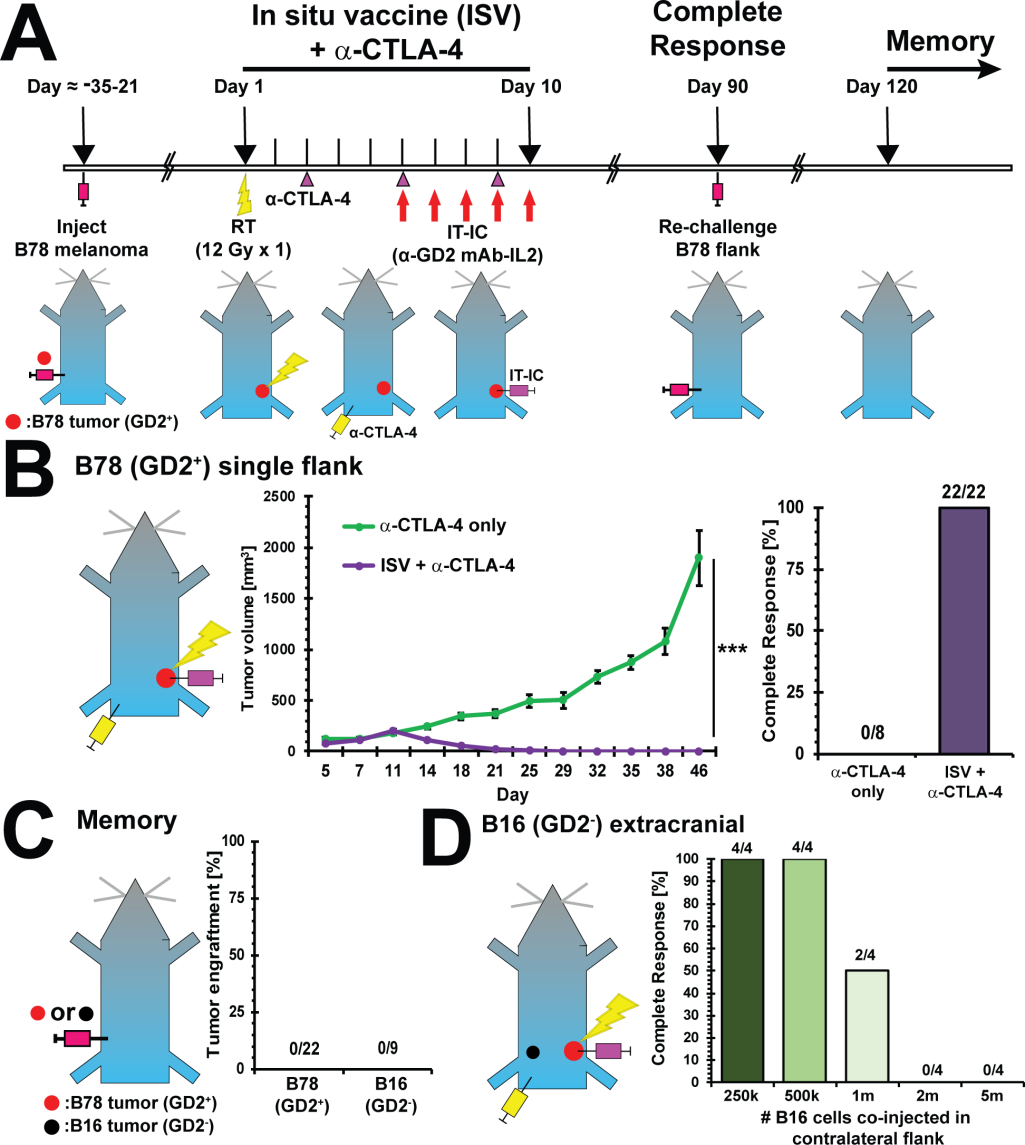
ISV + α-CTLA-4 regimen eliminates melanoma and establishes immune memory. (A) Experimental timeline for ISV + α-CTLA-4 regimen. (B) Tumor growth curve for immunologically ‘cold’ B78 (GD2^+^) melanoma treated with ISV + α-CTLA-4 regimen compared with α-CTLA-4 alone (mean±SE, n≥8, at least two independent animal experiments, ***p<0.001 comparing tumor growth rates, see also online supplementary figure 2), and complete responses (‘N’ above bars). (C) Tumor engraftment in ISV + α-CTLA-4 treated disease-free mice after rechallenge with B78 (2×10^6^ cells) or B16 (5000 cells, GD2^-^, parental line of B78) to test immune memory (‘N’ above bars). (D) Tumor response of B16 tumor coinjected in contralateral flank of mice with ISV + α-CTLA-4 treated primary B78 tumors (‘N’ above bars). IC, immunocytokine; IL-2, interleukin-2; IT, intratumor; mAB, monoclonal antibody.

An ISV + α-CTLA-4 regimen previously reported by our group was used to induce long-term melanoma tumor regression and immune memory (figure 1A).^5 30^ Briefly, B78 (GD2^+^) ‘primary’ tumors were engrafted by intradermal right flank injection of 2×10^6^ cells. Tumor size was determined using calipers and volume approximated as (width^2^ ×length)/2. Mice were randomized immediately prior to treatment. Treatment began when primary tumors were well-established (75–200 mm^3^ tumor volume). Radiation (12 Gy, single fraction) administration was defined as ‘day 1’ of treatment. IT-IC injections were made by a single percutaneous needle puncture followed by injection of 50 µg of hu14.18-IL2 in 100 µL volume with needle redirection to distribute injected material around the tumor. IT-IC was administered daily on treatment days 6–10. α-CTLA-4 antibody was administered by 200 µg IP injections on treatment days 3, 6 and 9.

10.1136/jitc-2020-000809.supp1 Supplementary data

Therapeutic ‘complete response’ (CR) was defined as mice having no remaining visible tumor at treatment day 90. In complete responders, immune memory was tested by engrafting 2×10^6^ B78 cells or 5000 B16 cells into the contralateral (left) flank and observing for any tumor growth with no follow-up treatments. If no palpable engrafted tumor was observed by 30 days later (treatment day 120), mice were considered to have developed immune memory. We have previously demonstrated that this memory response is T cell dependent.^5^

### Orthotopic brain injection

Intracranial implantation of cancer cells was performed as previously described.^31 32^ B78 or B16 cells were injected intracranially ~24 hours prior to irradiation of a pre-existing flank tumor. Briefly, cancer cells were enzymatically dissociated to single cells and varying cell numbers (2×10^5^ B78 cells or 200 B16 cells) were suspended in 5 µL of cell culture medium. Using a Hamilton syringe, the cells were stereotactically injected into the right striatum of anesthetized C57BL/6 mice at 1 µL/min at the following coordinates referenced from bregma: 0 mm antero–posterior, +2.5 mm mediolateral and −3.5 mm dorsoventral. The number of cells engrafted in the brain was optimized through serial dilution studies for B16 and B78 melanoma to determine the amount needed to consistently yield tumors while doing so over a time frame that could allow for interval development of an immune response (online supplementary figure 1). At specific time points or at onset of neurological symptoms, tumor-bearing mice were euthanized, and brains excised and processed for further analyzes.

### Multisite tumor experiments

For multisite experiments comparing extracranial to intracranial tumor responses, B78 (GD2^+^) cells (2×10^6^) were first implanted subcutaneously in the (right) flank of mice to initiate ‘primary’ tumor. The ‘primary’ tumor was allowed to grow for 2–3 weeks, at which point an extracranial distant ‘secondary’ tumor was injected into contralateral (left) flank, B78 (GD2^+^) at 2×10^6^ cells. An additional 1–7 days of growth was then allowed prior to treatment, as previously described.^30^ Contralateral (left) flank tumors were shielded from radiation treatment using lead.

To test immune recognition of antigens additional to targeted GD2 via epitope spreading, B16 (GD2^-^) cells at varying numbers (2.5×10^5^–5×10^6^) were injected into contralateral (left) flank when ‘primary’ B78 tumor treatment was started (75–200 mm^3^ tumor volume at ~4 weeks), as previously described.^5^

To analyze microenvironment effects of ISV + α-CTLA-4 at flank and brain tumors not directly treated, a ‘primary’ B78 tumor was first injected subcutaneously in the (right) flank and allowed to grow for 2–3 weeks. At that point, B78 tumors were injected both into contralateral (left) flank (2×10^6^ cells) and brain (2×10^5^ cells). After 15 days additional growth, treatment with IC, radiation, and α-CTLA-4 was started as above with radiation delivered on treatment ‘day 1’. At treatment day 15, mice were euthanized. All tumors (primary right flank, secondary left flank (LF) and brain tumor) and control contralateral normal brain were removed for analysis. Tissue was processed for immunohistochemistry (IHC), flow cytometry, ELISA and quantitative (q)RT-PCR.

### Flow cytometry

Flow cytometry was used to analyze immune cell populations^5 33^ in a minimum of three samples in two independent animal studies, with matched multisite tissue from each animal that included primary tumor site (right flank), contralateral secondary extracranial site (LF), intracranial brain melanoma and contralateral normal brain. Dissected tissue and tumors were enzymatically dissociated to single cell using collagenase and DNAase along with mechanical shaking at 37°C. Tissue slurry was then filtered through a 70 µm cell filter. Single cells were labeled with primary antibody. Cells without primary antibody labeling were used as unlabeled negative controls; fluorescent beads (UltraComp Beads eBeads, Invitrogen, #01-2222-42) were used as positive/calibration controls and to determine compensation between fluorescent channels. Forward and side-scatter gating identified single cells and viable cells (Ghost Dye Red 780 Viability Dye, 1:100, Tonbo Biosciences, #13–0865 T100) exclusion identified live cells. Fluorescence minus one methodology was used to determine gating. Flow cytometry was performed on an Attune NxT Flow Cytometer (ThermoFisher), and compensation matrix computed and data analyzed using FlowJo (v9) software following published flow cytometry guidelines.^34^

Antibodies used: CD4 (BV510, 1:400, Biolegend, clone RM4-5, #100553), CD8 (PerCP-cy5.5, 1:200, Biolegend, clone 53–6.7, #100734), FOXP3 (BV421, 1:100, Biolegend, clone MF-14, #126419), CD45 (PE-cy7, 1:200, Tonbo Biosciences, clone 30-F11, #60–0451 U100), CD3 (FITC, 1:200, Tonbo Biosciences, clone 17A2, #35–0032 U100), CD25 (APC, 1:200, Biolegend, clone PC61, #102011).

### Immunohistochemistry

For IHC sections, antibodies used were: CD4 (mAb Rat IgG2b, kappa; clone GK1.5; Tonbo Biosciences 70–0041 U500l, 1:1000 dilution), CD8a (mAb Rat IgG2a, kappa; clone 53–6.7; eBioscience 14-0081-85, 1:1000), FOXP3 (mAb Rat IgG2a, kappa; clone FJK-16S; eBioscience 14-5773-82, 1:500), F4/80 (mAb Rat IgG2a,κ; clone BM8; BioLegend 123101, 1:2000), CD11b (mAb Rat, clone M1/70.15, Invitrogen MA5-17857, 1:6000); specific for paraffin were CD4 (mAb Rat IgG_1_, kappa; clone 4SM95; eBioscience 14-9766-82, 1:500) and CD8a (mAb Rat IgG2a, lambda; clone 4SM15; eBioscience 14-0808-80, 1:250). Standard IHC methods were performed as previously described.^5 35^ All labeling was performed with no primary antibody negative controls. A minimum of three random high-power fields per tumor sample were quantified for positive labeling by a blinded observer, with serial slides of H&E used to determine viable tumor areas. For F4/80 immunolabeling, individual labeled cells were difficult to distinguish and therefore percentage labeled area was calculated using multiple color-balanced 20X fields and ImageJ ‘Color Deconvolution (H DAB)’, followed by thresholding of the deconvoluted 3,3’-Diaminobenzidine (DAB) image. The multiple high-power fields were averaged for each individual tumor sample (ie, mouse) to achieve a single ‘n’ for statistical purposes; results were charted as mean±SE of the mean with data points representing individual mice (‘n’).

### Quantitative reverse transcription-PCR

For analysis of tumor tissue, tumor samples were homogenized using a Bead Mill Homogenizer (Bead Ruptor Elite, Omni International). Total RNA was extracted after sample homogenization using RNeasy Mini Kit (QIAGEN) according to the manufacturer’s instructions. Extracted RNA was subjected to complementary cDNA synthesis using QuantiTect Reverse Transcription Kit (QIAGEN) according to the manufacturer’s instructions. qRT-PCR was performed using PowerUp SYBR Green qPCR Master Mix. The reaction (5 µL total volume) was prepared using Labcyte Echo 550 and MANTIS liquid handling systems. Thermal cycling conditions (Vii7A Cycler, Applied Biosystems) included the Uracil-DNA N-glycosylase (UDG) activation stage at 50°C for 2 min, followed by Dual-Lock DNA polymerase activation stage at 95°C for 2 min followed by 40 cycles of each PCR step: (denaturation) 95°C for 15 s and (annealing/extension) 60°C for 1 min. A melt curve analysis was also done to ensure the speciﬁcity of the corresponding qRT-PCR reactions. For data analysis, the Ct values were exported to an Excel ﬁle and fold change was calculated using the ∆∆Ct method. *Hprt*, *Pgk1* and *Tbp* were used as endogenous controls. The following primers were used: *Pd-l1*: f: ATG TCA GGC CGA GGG TTA TC, r: TCT CTT CCC ACT CAC GGG TT; *Mhc-1*: f: GTA CCA TCG CAC CTG TCG G, r: CCG CGG ACG CTG GAT ATA AA; *Cd80*: f: ACT ACC CTG GCT CTG CAA AC, r: CGT CCT CAG AAT CAG AAT CAG CAG AAC T; *Tigit*: f: GAA GCC CTG TCC AGA CAC AA, r: TTC CTG TGG GTC AGC ATA GTC; *Ctla-4*: f: ACC TCT GCA AGG TGG AAC TC, r: AAG TCA GAA TCC GGG CAT GG; *Lag3*: f: CAG CTC AAT GCC ACT GTC AC, r: TTT CCA GAT GCC GGG GTT AC; *Hprt*: f: AGC CTA AGA TGA GCG CAA GT, r: GGC CCA CAG GAC TAG AAC ACC; *Pgk1*: f: GGC ATT CTG CAC GCT TCA AA, r: CGA CAT TTT GGC AAC ACC GT; *Tbp1*: f: GTT GGG CTT CCC AGC TAA GT, r: CAC AAG GCC TTC CAG CCT TA.

### Tumor cytokine multiplex immunoassay

Tumor weight was recorded and 5 µL/mg of Cell Lysis Buffer with PMSF (Cell Signaling Technology) and Halt Phosphatase Inhibitor Cocktail (Thermo Scientific) was added to the tumor. Tumors were homogenized in bead beater tubes, and the lysates were stored at −80°C until use. A multiplex immunoassay was used to determine the concentration of 32 cytokines and chemokines in the tumor lysates (MILLIPLEX MAP Mouse Cytokine/Chemokine Magnetic Bead Panel, Millipore) following the manufacturer’s instructions. The multiplex was read on the MAGPIX System (Millipore), and the protein concentrations were interpolated from curves constructed from the protein standards and their respective median fluorescence intensity readings (Milliplex Analyst, Millipore). Log and Z-transformation of the data was performed using SPSS V.25 (IBM) and followed by unbiased hierarchical clustering (clustering only cytokines or both animals and cytokines) using on-line tool (Next Generation Clustered Heat Maps; MD-Anderson Center, University of Texas, http://www.ngchm.net/; Euclidean distance metric and McQuitty Agglomeration; accessed January 13 2020,^36^).

### Statistical analyses

All results are displayed as mean±SE of the mean, unless otherwise noted. Tumor volume was summarized by geometric mean and SD and the tumor growth rates of treatment groups were plotted on logarithmic scale, using R (V 3.6.2). The group difference in log-transformed tumor growth was assessed via linear mixed-effects model using ‘proc glimmix’ in SAS V.9.4. Survival curves were analyzed using the Kaplan-Meier method; Benjamini & Hochberg’s method for p values adjustments was used to assess multiple comparisons of survival curves. Student’s t*-*test was used for two-sample comparisons. Analysis of variance (ANOVA) and Bonferroni’s method for p values adjustments were used to assess the multiple comparison among groups. P values less than 0.05 were considered significant and are indicated in figures as ***p<0.001; **p<0.01; *p<0.05; NS: non-significant (p≥0.05). Survival, two-sample, and multiple comparison analyzes were performed using IBM SPSS V.25 or Graphpad Prism V.8 software. All statistical tests were two sided with p values less than 0.05 considered statistically significant.

## Results

### ISV augments response to ICI

Using a previously optimized regimen,^5^ we tested the ability of our combined modality ISV + α-CTLA-4 treatment to eliminate B78 GD2^+^ melanoma tumors on the flank and establish immune memory. For this, we engrafted ‘primary’ B78 tumors on the right flank and 3–4 weeks later mice were treated with radiation (12 Gy, day 1), IC (hu14.18-IL2, 50 µg IT injected daily, days 6–10) and α-CTLA-4 ICI (200 µg IP injected, days 3, 6 and 9)^5 30^ (figure 1A). Primary established flank tumors were treated with this ISV + α-CTLA-4 regimen (mean±SE starting tumor volume=87±6.8 mm^3^) or α-CTLA-4 alone (mean±SE starting tumor volume=89±7.0 mm^3^). Following ISV + α-CTLA-4 treatment, all B78 flank tumors regressed in volume and all mice remained alive with CR at day 90 (n=22). In contrast with this immunologically ‘cold’ B78 melanoma model, tumors in control mice treated only with α-CTLA-4 continued to grow and no CRs were observed (n=8) (figure 1B). Flank tumor growth analysis demonstrated an average mouse receiving ISV had an additional 20% decrease (95% CI 18% to 21% reduction, p<0.001) in geometric mean of tumor volume for each additional day of treatment, as compared with an average mouse receiving α-CTLA-4 alone (online supplementary figure 2). Consistent with the development of immunologic memory, all ISV + α-CTLA-4 treated complete responders rejected B78 as well as B16 tumor re-engraftment in the contralateral flank (n=22) (figure 1C).

To test whether this ISV + α-CTLA-4 regimen would induce an abscopal response against tumor cells present at the time of treatment but not directly targeted by radiation or IT-IC, we engrafted mice with a primary B78 melanoma tumor on the right flank. On treatment day 1, we engrafted the contralateral LF with B16 melanoma. B16 is parental to and shares common antigens with B78 but lacks the GD2 antigen targeted by hu14.18-IL2 IC. ISV + α-CTLA-4 treated mice eliminated these contralateral B16 tumors even when up to 1 million cells were injected (figure 1D).

### Mice developing immune memory from ISV reject intracranial melanoma

**Figure 2:**
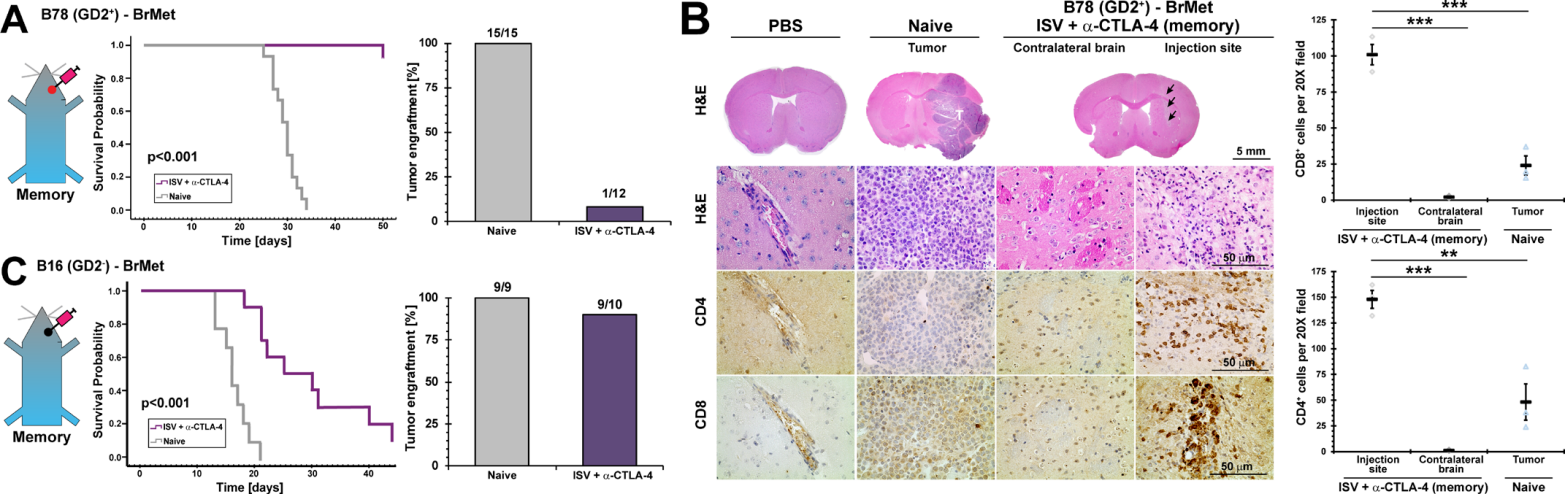
Extracranial established immune memory is conveyed to central nervous system. Immune memory in mice was established by treating primary extracranial B78 melanoma with ISV + α-CTLA-4 regimen. (A) Survival curve for immune memory mice receiving intracranial injection of B78 (p<0.001, Kaplan-Meier, n≥12, at least two independent animal experiments), and successful brain tumor engraftments (‘N’ above bars). (B) Immunohistochemistry representative images of immune memory mouse brain 4 weeks after receiving B78 intracranial injections, compared with naïve receiving B78 intracranial and PBS sham-injected controls (arrows: injection track, brown=positive immunolabeling, T: tumor, H&E) and quantified IHC (**p<0.01, ***p<0.001, mean±SE with marker representing each individual mouse (ie, average of three high-powered fields), ANOVA with post hoc Bonferroni, n≥3). (C) Survival curve for immune memory mice receiving intracranial injection of B16 (GD2^-^) (p<0.001, Kaplan-Meier, n≥9, at least two independent animal experiments), and successful brain tumor engraftments (‘N’ above bars). ANOVA, analysis of variance; BrMet, brain met; IHC, immunohistochemistry; ISV, in situ vaccination.

To test whether immune memory induced by ISV + α-CTLA-4 at an extracranial site was effective in preventing tumor engraftment in the brain, we engrafted mice with B78 melanoma on the right flank and rendered them disease free as above. At day 90 after treatment initiation, we verified the presence of a memory response by confirming rejection of re-engraftment with B78 melanoma in the LF (as in figure 1A). At day 120, after confirming immune memory, we stereotactically implanted B78 melanoma (200 000 cells) into the right striatum of the mouse brain. Whereas all naïve controls (n=15) developed melanoma in the brain and died within 35 days after B78 engraftment, 11 of 12 mice previously rendered disease-free by ISV + α-CTLA-4 rejected intracranial B78 melanoma engraftment, resulting in prolonged survival (p<0.001) (figure 2A). For the 1 (of 12) mouse that failed to reject brain implanted B78 melanoma cells, we observed an increased number of CD8^+^ and CD4^+^ T-cells compared with brain tumors in naïve mice (online supplementary figure 3).

In a separate cohort, mice were euthanized at day 28 after intracranial injection to evaluate tumor growth and brain tumor immune infiltrate by IHC. No tumors were identified by histology (H&E) in mice with previously established B78 immune memory and receiving B78 intracranial injections, compared with all treatment-naïve mice developing brain tumors (figure 2B). Significantly increased CD8^+^ and CD4^+^ T-cells were detected at the melanoma injection site even though tumor cells were no longer observed, compared with contralateral brain parenchyma and naïve mice (for CD8^+^, injection site: 101±7.0 cells per 20X field, for contralateral brain: 2.0±0.5, and naïve mice: 24±6.6; for CD4^+^, injection site: 150±8.7 cells per 20X field, for contralateral brain: 1.1±0.67, and naïve mice: 48±18; mean±SE, n≥3).

We further evaluated whether ISV + α-CTLA-4-induced immune memory could reject brain engraftment of the more aggressive parental B16 (GD2^-^) melanoma cells. For this, we stereotactically injected B16 melanoma cells (200 cells) into brain as above. ISV + α-CTLA-4-induced immune memory significantly increased survival compared with control naïve mice (p<0.001). However, only 1 of 10 ISV + α-CTLA-4 treated mice completely rejected engraftment of the more invasive and rapidly growing B16 tumor cells in the brain, verified by dissection and gross examination of brain at endpoint to confirm no gross tumor was present (B16 tumors are black and readily identified at the injection site when present) (figure 2C).

### Melanoma present in the brain at the time of ISV treatment exhibit minimal response

**Figure 3:**
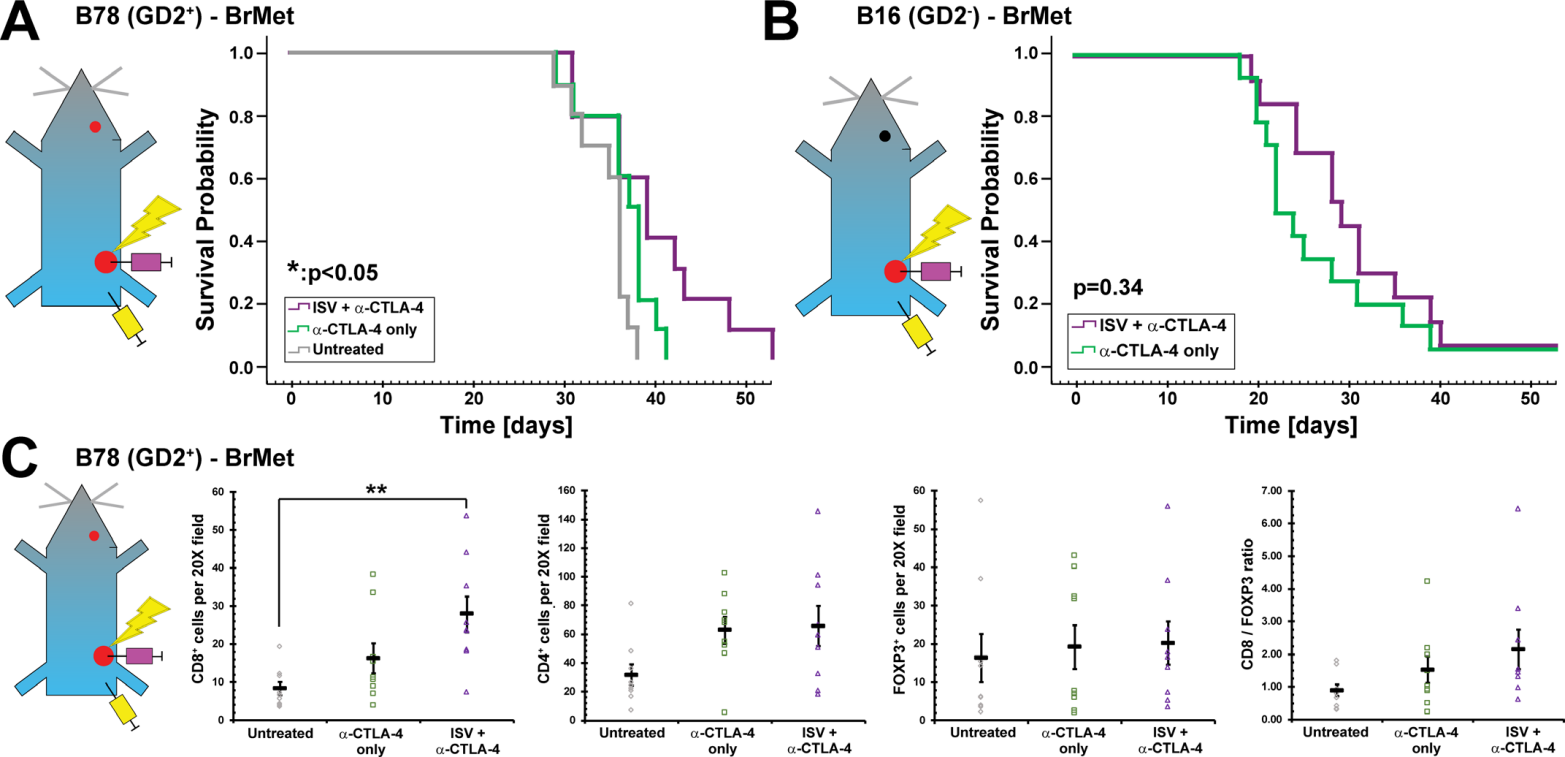
ISV + α-CTLA-4 regimen against extracranial melanoma increases survival for mice harboring intracranial tumors. (A) Survival curves for mice receiving intracranial B78 injections ~24 hours prior to initiation of ISV + α-CTLA-4 regimen against extracranial B78 tumor (*p<0.05, Kaplan-Meier, n=10, at least two independent animal experiments). (B) Survival curves for mice receiving intracranial B16 injections ~24 hours prior to initiation of ISV + α-CTLA-4 regimen against extracranial B78 tumor (p=0.34, Kaplan-Meier, n≥13, at least two independent animal experiments). (C) Immunohistochemistry quantified for B78 intracranial tumors at death for mice that had received ISV + α-CTLA-4 regimen against extracranial B78 tumor (**p<0.01, mean±SE with marker representing each individual mouse (ie, average of three high-powered fields), ANOVA with post hoc Bonferroni, n=9, at least two independent animal experiments). ANOVA, analysis of variance; BrMet, brain met; ISV, in situ vaccination.

To model metastases present in the brain before starting ISV + α-CTLA-4 treatment targeting an extracranial tumor, we engrafted mice with a primary right flank B78 tumor, allowed this to grow for 3–4 weeks, then engrafted B78 melanoma (200 000 cells) in the right striatum of the brain 24 hours prior to initiation of ISV + α-CTLA-4 treatment. Mice were treated as above with α-CTLA-4 and ISV + α-CTLA-4 targeting the right flank tumor and were compared with untreated mice or those receiving only α-CTLA-4. ISV + α-CTLA-4 significantly increased survival of mice bearing B78 brain tumors compared with untreated mice (p<0.05, n=10), but the addition of ISV to α-CTLA-4 did not significantly improve survival compared with α-CTLA-4 alone (figure 3A). Similarly, mice engrafted with B16 melanoma in the brain 24 hours prior to ISV + α-CTLA-4 treatment demonstrated no improvement in survival (p=0.34, n≥13), compared with mice treated with α-CTLA-4 only (figure 3B). B78 melanoma brain tumors in ISV + α-CTLA-4 treated mice at death were analyzed by IHC to evaluate immune infiltrate. CD8^+^ T cells were significantly increased in ISV + α-CTLA-4 treated mice (28±4.7 cells per 20X field, mean±SE, p<0.05, ANOVA with post hoc Bonferroni, n=9), compared with untreated mice (8.3±1.7 cells per 20X field) but not compared with α-CTLA-4 alone (16±4.0 cells per 20X field) (figure 3C). CD4^+^ and FOXP3^+^ T cells, as well as the ratio of CD8^+^:FOXP3^+^ T cells trended toward an increase in ISV + α-CTLA-4 treated mice compared with other groups but this was not statistically significant (for CD4^+^, ISV + α-CTLA-4: 70±23 cells per 20X field, α-CTLA-4 alone: 57±14, untreated: 36±13; for FOXP3^+^, ISV + α-CTLA-4: 20±5.6, α-CTLA-4 alone: 19±5.7, untreated: 16±6.3; for CD8^+^:FOXP3^+^ ratio, ISV + α-CTLA-4: 2.15±0.61, α-CTLA-4 alone: 1.5±0.40, untreated: 0.90±0.18; mean±SE, n=9) (figure 3C).

### Reduced infiltration of CD8^+^ T cells in brain versus flank melanoma following ISV

**Figure 4:**
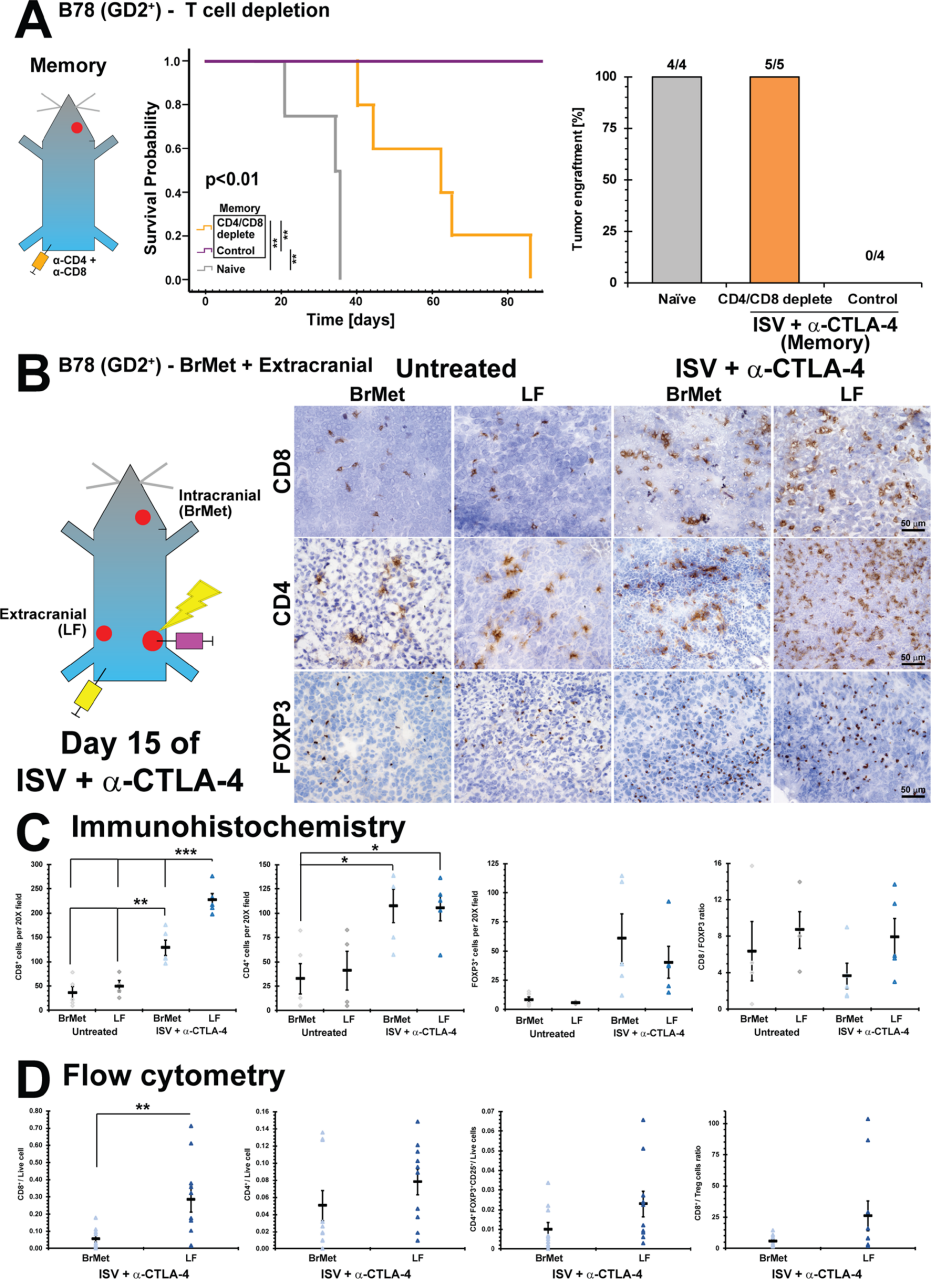
Intracranial versus extracranial infiltrating immune cell analysis after ISV + α-CTLA-4 treatment. (A) Survival curve for immune memory mice depleted of T-cells (CD4 and CD8) prior to receiving intracranial injection of B78 (**p<0.01, Kaplan-Meier, n≥4) compared with control (rat IgG) and naïve, and successful brain tumor engraftments (‘N’ above bars). (B) Immunohistochemistry of ISV + α-CTLA-4 treated and untreated mice, comparing intracranial (brain met, BrMet) and extracranial (left flank, LF) B78 melanoma tumors (brown=positive immunolabeling). (C) Quantified immunohistochemistry (***p<0.001, **p<0.01, *p<0.05, mean±SE with marker representing each individual mouse (ie, average of three high-powered fields), ANOVA with post hoc Bonferroni, n≥5, at least two independent animal experiments). (D) Flow cytometric analysis of B78 tumors after ISV + α-CTLA-4 treatment comparing BrMet to extracranial tumors (**p<0.01, mean±SE with marker representing individual data points, ANOVA with post hoc Bonferroni, n≥8, at least two independent animal experiments). ANOVA, analysis of variance; ISV, in situ vaccination.

We hypothesized that mice rendered disease-free by ISV + α-CTLA-4 rejected intracranial re-engraftment with melanoma because of an adaptive antitumor T cell memory response. We tested the necessity of T cells for rejecting melanoma brain metastases through a depletion experiment. CD4^+^ and CD8^+^ T cells were depleted by IP injection of α-CD4 and α-CD8 antibodies in mice with verified extracranial immune memory following CR to ISV + α-CTLA-4 treatment. These α-CD4 and α-CD8 antibodies deplete >97% of respective T-cells in the blood by 7 days after initial administration (online supplementary figure 5). Mice were then engrafted with B78 in the right striatum of the brain. While ISV + α-CTLA-4 treated mice consistently rejected intracranial B78 engraftment, all naïve mice and all mice depleted of CD4^+^ and CD8^+^ T cells exhibited B78 tumors in the brain, verified by dissection and gross examination at moribund or day 90, respectively (figure 4A). This is consistent with ISV + α-CTLA-4 leading to T cell-dependent immune memory against melanoma brain metastases. Although brain tumors did develop in mice with tumor-specific immunologic memory receiving α-CD4 and α-CD8 depleting antibodies, survival was significantly longer than naïve mice (p<0.01, figure 4A), suggesting additional non-T cell dependent mechanisms of immune memory against metastatic tumors in the brain.

We next evaluated whether immune cell response might correlate with differences in the capacity of ISV + α-CTLA-4 to elicit antitumor responses for extracranial versus intracranial melanomas. Mice with established B78 melanoma primary tumors (right flank) were implanted with extracranial (LF) and intracranial (brain met, BrMet) B78 tumors 15 days prior to ISV treatment. Mice were treated as above with ISV + α-CTLA-4 and then euthanized at day 15 after initiation of ISV. Immune infiltrate was evaluated by IHC and by flow cytometry on disaggregated tumors. We previously established day 15 post-ISV as an approximate time of peak T-cell response.^5^

Using IHC, we observed increased infiltration by T cells in tumors of ISV + α-CTLA-4 treated mice compared with untreated controls, regardless of tumor location (figure 4B). This is consistent with successful ISV stimulation of a more robust systemic antitumor immune response. However, among ISV + α-CTLA-4 treated mice we observed significantly increased CD8^+^ T cell infiltration in the extracranial LF tumor compared with the intracranial brain tumor (p<0.05 by ANOVA with post hoc Bonferroni; for ISV + α-CTLA-4 treated mice - LF: 230±13 per 20X field, BrMet: 130±16; for untreated mice - LF: 49±11, BrMet: 36±13; mean±SE, n≥5) (figure 4C). Significantly higher CD4^+^ T cells were also observed in tumors of ISV + α-CTLA-4 treated mice compared with untreated mice, regardless of tissue location (p<0.05 by ANOVA with post hoc Bonferroni; for ISV + α-CTLA-4 - LF: 106±13 per 20X field, BrMet: 107±17; for untreated - LF: 41±20, BrMet: 32±16; mean±SE, n≥5) (figure 4B). However, no significant differences were observed in tumor infiltration by FOXP3^+^ T cells, though a non-significant trend was observed toward an increase in tumors from ISV + α-CTLA-4 treated mice (for ISV + α-CTLA-4 - LF: 40±14 per 20X field, BrMet: 61±21; for untreated - LF: 5.8±0.28, BrMet: 8.2±2.5; mean±SE, n≥5) (figure 4B).

We further analyzed immune cell infiltration in extracranial and intracranial B78 melanoma tumors using flow cytometry. Our gating strategy is presented in online supplementary figure 4. Corroborating IHC findings, significantly increased CD8^+^ T cells were found in extracranial (LF) compared with intracranial (BrMet) tumors in ISV + α-CTLA-4 treated mice (p<0.05, LF: 0.29±0.075 CD8^+^/live cell, BrMet: 0.056±0.019; mean±SE, n≥8) (figure 4D). No significant differences were observed among CD4^+^ or Treg (CD4^+^FOXP3^+^CD25^+^) populations, although trends supported observations from IHC (figure 4D).

To further interrogate the role of Tregs in melanoma brain tumor progression, we used transgenic C57BL/6 ‘DEREG’ mice in which the diphtheria toxin (DT) receptor is expressed downstream of the FoxP3 promoter, resulting in constitutive expression of this receptor on Tregs. This transgenic model enables depletion of Tregs on treatment with DT.^27^ We first validated Treg depletion and observed >40% knockdown by day 3 (1 day after completion of two DT injections of 1 µg) (online supplementary figure 6), with previous reports indicating >90% depletion by day 7.^27^ Our previous work has demonstrated significant reduction in tumor growth at both primary and distant extracranial melanoma sites after elimination of Tregs using this approach.^21^ When DT was administered to mice harboring a melanoma primary and intracranial tumor and treated with or without ISV to the primary tumor, no significant survival benefit was measured (p=0.858, online supplementary figure 6). Gross examination at euthanasia also verified brain tumor in all mice, suggesting no confounding effect of Treg depletion on autoimmunity and subsequent mortality.

**Figure 5:**
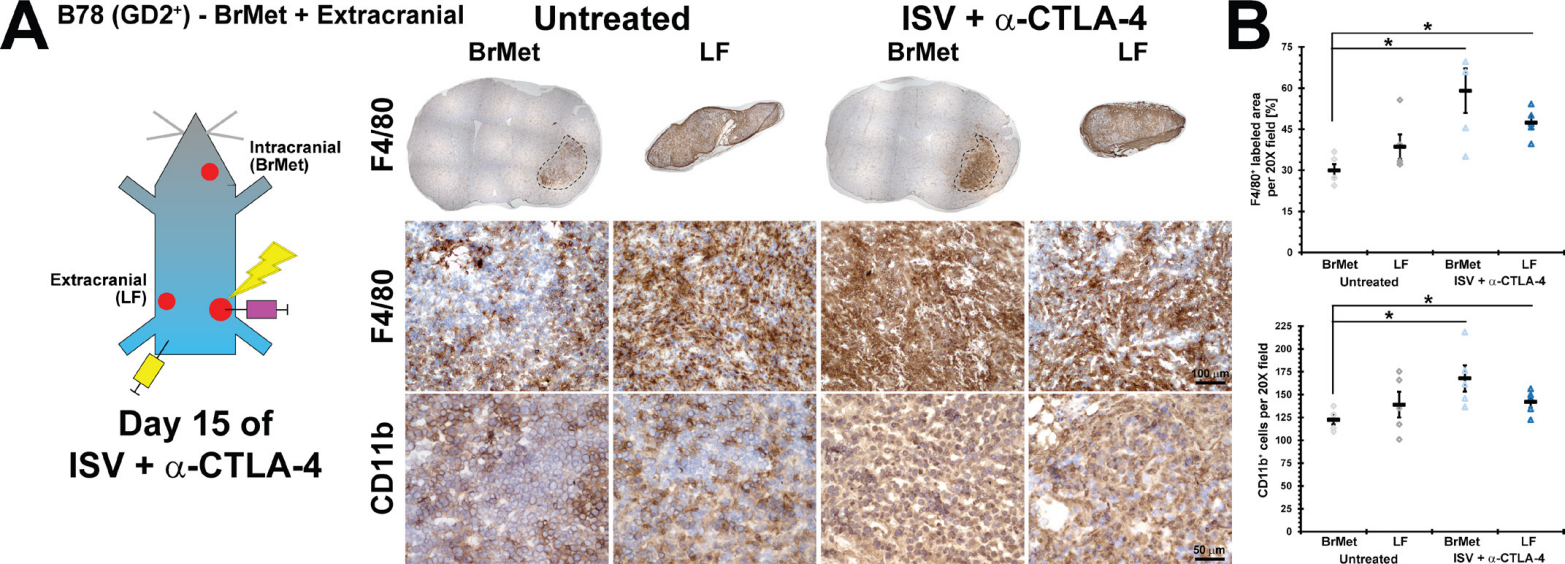
Intracranial versus extracranial myeloid and monocytic/macrophage immune cell analysis after ISV + α-CTLA-4 treatment. (A) Immunohistochemistry of ISV + α-CTLA-4 treated and untreated mice, comparing intracranial (brain met, BrMet) and extracranial (left flank, LF) B78 melanoma tumors (brown=positive immunolabeling; dotted line is brain tumor determined using hematoxylin). (B) Quantified immunohistochemistry (*p<0.05, mean±SE) with marker representing each individual mouse (ie, average of three high-powered fields from the indicated tumor specimen), ANOVA with post hoc Bonferroni, n≥5, at least two independent animal experiments). ANOVA, analysis of variance; ISV, in situ vaccination.

We performed IHC using innate immune lineage markers of myeloid cells and monocytes/macrophages on brain tumors from untreated mice compared with ISV + α-CTLA-4 treated mice. We observed a significant increase in the number of cells staining for the myeloid marker CD11b in ISV+α-CTLA-4 treated intracranial tumors compared with those from untreated mice, without a comparable change in the peripheral tumors from these mice (figure 5A, for ISV + α-CTLA-4 - LF: 140±6 labeled cells per 20X field, BrMet: 170±14; for untreated - LF: 140±14 labeled cells per 20X field, BrMet: 120±5.0; mean±SE, n≥5). Similar results were obtained for the monocyte/macrophage marker F4/80 (% area of 20X field labeling for ISV + α-CTLA-4 - LF: 47±2.4, BrMet: 59±8.1; for untreated - LF: 39±4.5, BrMet: 30±2.3; mean±SE, n≥5) (figure 5B). This suggests a potential role for these non-T-cell immune lineages in the response and suppression of antitumor immunity in the brain following ISV + α-CTLA-4.

### Expression of immune susceptibility genes and cytokines in brain versus flank melanoma

IHC and flow cytometry demonstrated that ISV stimulates a more effective systemic antitumor response that results in increased tumor infiltrate by CD8^+^ T cells. Even among melanoma tumors in the brain, we observed a marked increase in tumor infiltrating T cells compared with untreated mice, although this response was greater in flank tumors. To evaluate why this apparent immune response was effective in eradicating extracranial tumors but ineffective in eradicating tumors in the brain, we evaluated the expression of immune checkpoint receptors/ligands and markers of tumor cell immune susceptibility in the brain and flank tumor microenvironment.

**Figure 6:**
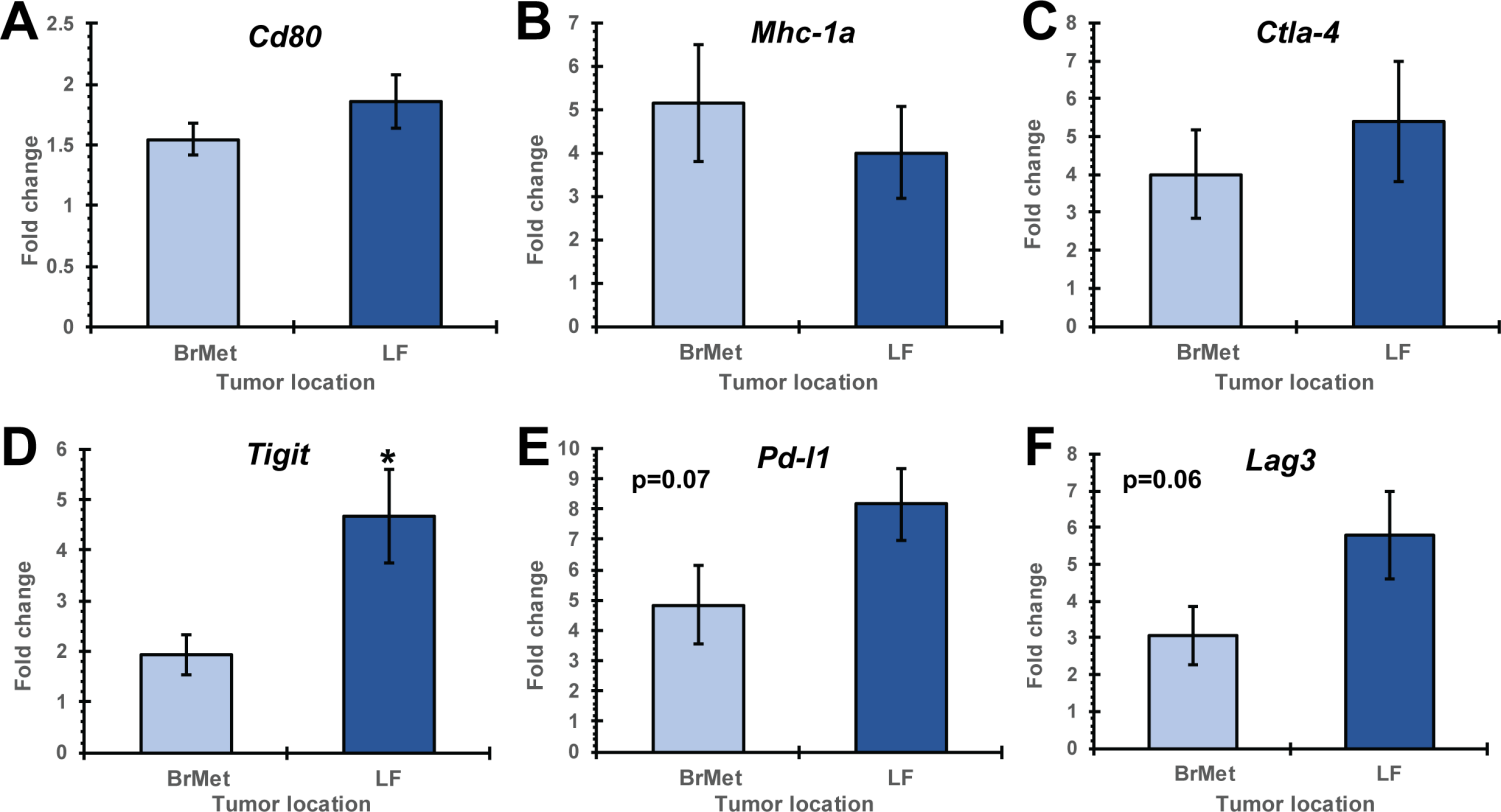
qRT-PCR analysis of ISV + α-CTLA-4 treated mice comparing intracranial (BrMet) to extracranial (LF) B78 tumors. Immune checkpoint receptor expression of *Cd80* (A), *Mhc-1a* (B), *Ctla-4* (C), *Tigit* (D), *Pd-l1* (E) and *Lag3* (F) shown as fold change to untreated controls (BrMet, LF (extracranial secondary tumor); *p<0.05 or p values listed, mean±SE, Student’s t-test, n≥14, three independent animal experiments). BrMet, brain met; ISV, in situ vaccination; LF, left flank.

As in figure 4, mice with established B78 melanoma primary tumors (right flank) were implanted with extracranial (LF) and intracranial (BrMet) B78 tumors 15 days prior to ISV + α-CTLA-4 treatment. Mice were treated as above and then euthanized at day 15 after initiation of ISV. Tumors were dissected and bulk mRNA was extracted. Using qRT-PCR, we observed minimal differences in expression of immune checkpoint receptors/ligands and markers of tumor cell immune susceptibility in B78 melanoma tumors from the LF and brain (figure 6). The inhibitory receptor *Tigit* was significantly increased in extracranial (LF) compared with intracranial (BrMet) tumors (p<0.05 by Student’s t-test, n≥14) (figure 6D). All other tested receptors were not significantly different including *Cd80*, *Mhc-1a*, and *Ctla-4*; although *Pd-l1* and *Lag3* trended toward increased expression in extracranial compared with intracranial tumors (p=0.07 and 0.06, respectively) (figure 6).

**Figure 7:**
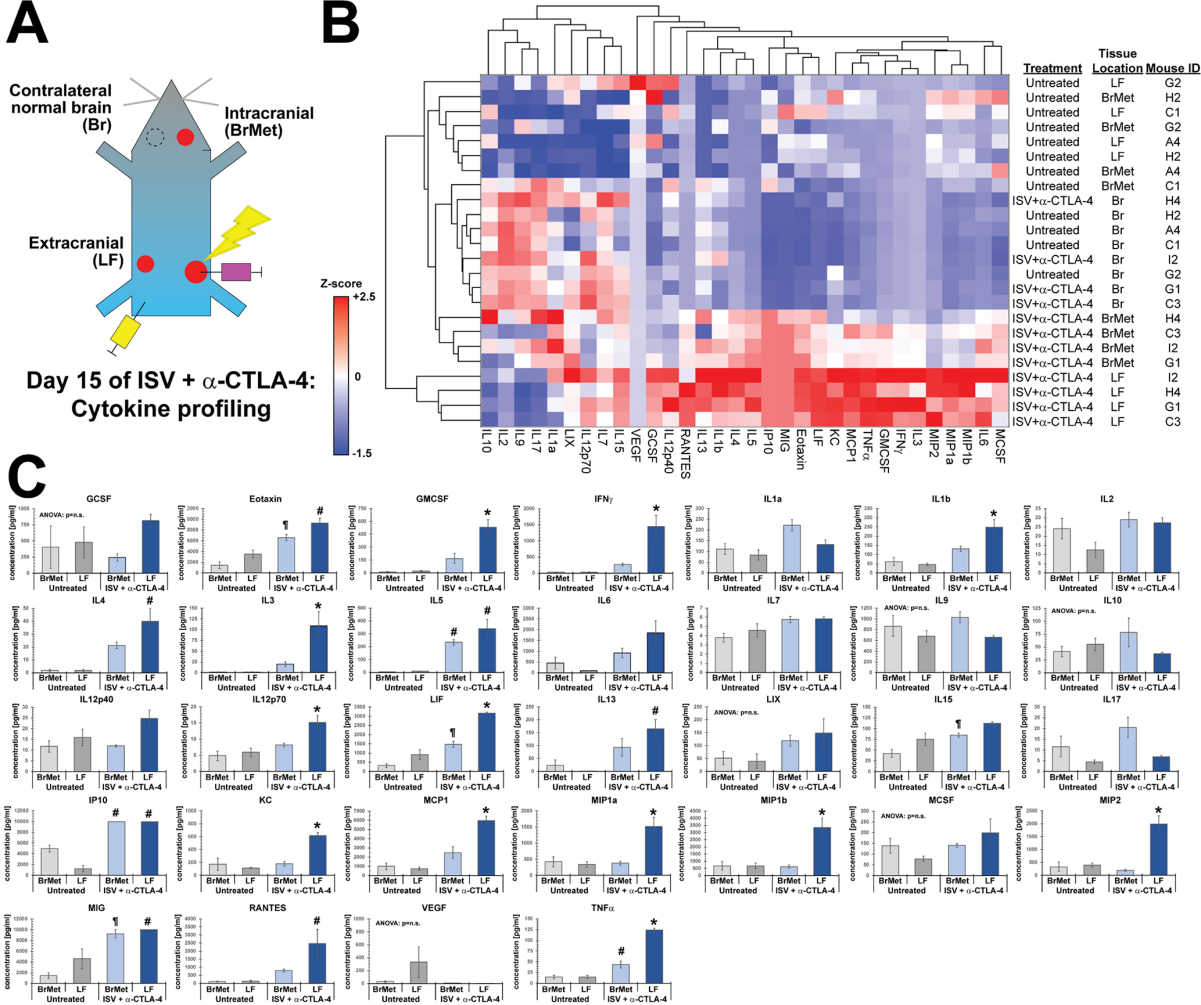
Cytokine/chemokine protein expression in ISV + α-CTLA-4 treated versus untreated mice. (A) Experimental schematic for tissue/tumor locations analyzed via multiplex ELISA, for ISV + α-CTLA-4 treated or untreated mice. (B) Heatmap of cytokines analyzed via multiplex ELISA hierarchically clustered by both treatment/tissue location and cytokine (z-scores; n=4 mice in a single animal experiment). BrMet: brain Met, LF: left flank (extracranial secondary tumor), Br: contralateral control brain. (C) Individual cytokine/chemokine concentrations from (B) (one-way ANOVA, significant among groups p<0.05 unless indicated; followed by post hoc Bonferroni, *p<0.05 compared with all other tissue locations; #p<0.05 compared with untreated tumors (both locations); ¶p<0.05 compared with untreated brain Met; mean±SE, n=4 mice in a single animal experiment). ANOVA, analysis of variance; ISV, in situ vaccination.

Finally, using tumor fragments from these same mice, we analyzed the production of cytokines and chemokines in the microenvironment of tumors from the flank and brain of untreated and ISV + α-CTLA-4 treated mice. Multiplex cytokine assay was performed, and unbiased hierarchal clustering used to sort tumors based on detected levels of cytokines/chemokines. Interestingly, in untreated mice, tumors from flank and brain did not differ in the production of assayed cytokines or chemokines (figure 7). In contrast, ISV + α-CTLA-4 treated mice brain tumors clustered separately from LF tumors and differed considerably in the levels of many cytokines and chemokines (figure 7). Like results from IHC and flow cytometry, we observed a general pattern of decreased production of immune stimulatory cytokines in the brain tumor microenvironments compared with extracranial tumor microenvironments in ISV + α-CTLA-4 treated mice (figure 7). Particularly notable and significant differences were observed in the concentrations of several immune stimulatory cytokines and chemokines at extracranial compared with intracranial brain melanomas from ISV + α-CTLA-4 treated mice, including interferon-γ (IFN-ɣ), MIP-1a, MIP-1b, MIP-2, interleukin-1b (IL-1b), and granulocyte-macrophage colony-stimulating factor (GM-CSF) (figure 7C). In contrast, non-significant trends were observed toward increased production of the inhibitory cytokine IL-10, MDSC-activating IL-17 and the neuroinflammatory cytokine IL-1a in B78 tumors from the brain of ISV + α-CTLA-4 treated mice, compared with untreated controls and extracranial tumors from ISV + α-CTLA-4 treated mice (figure 7C).

## Discussion

Using an immunologically ‘cold’ murine model of melanoma that does not respond to immune checkpoint blockade alone, we tested whether an ISV therapeutic regimen of radiotherapy and IT injection of a tumor-specific IC at an extracranial tumor could augment the response to α-CTLA-4 immune checkpoint blockade at an intracranial tumor. In combination with α-CTLA-4, we observe that this ISV regimen effectively eradicates the targeted primary tumor site and also consistently eradicates distant non-targeted extracranial tumor sites resulting in the establishment of tumor-specific T cell memory^5^ (figure 1). We observe that this memory response is capable of preventing engraftment of melanoma tumor cells in the brain (figure 2). This suggests that once established, antitumor immunity generated by our ISV + α-CTLA-4 regimen can effectively kill tumor cells in the brain. However, when melanoma is present in the brain before or at ISV + α-CTLA-4 treatment, this regimen cannot inhibit tumor growth in the brain. The rate of tumor growth in brain and flank was observed to be similar for this melanoma model and ISV effectively eradicates extracranial tumor sites before mice succumb to brain tumor growth. This suggests that there is adequate time for the ISV regimen to stimulate an effective systemic antitumor response against intracranial tumor sites. In fact, by IHC and flow cytometry we observe an effect of ISV in significantly enhancing CD8^+^ T cell infiltration of melanoma tumors in both flank and brain tumors, even when these tumors are present in the brain at the time of ISV (figure 4). The inability of this cellular immune response to control melanoma tumor sites in the brain despite concurrent eradication of distant flank tumor sites could result from differences in the magnitude of immune response, as we observed significantly fewer CD8^+^ T cells and a decreased ratio of effector CD8^+^: suppressor Tregs in intracranial melanomas. These results demonstrate a clear and novel effect of tumor location on the propagation of an abscopal response to ISV.

**Figure 8:**
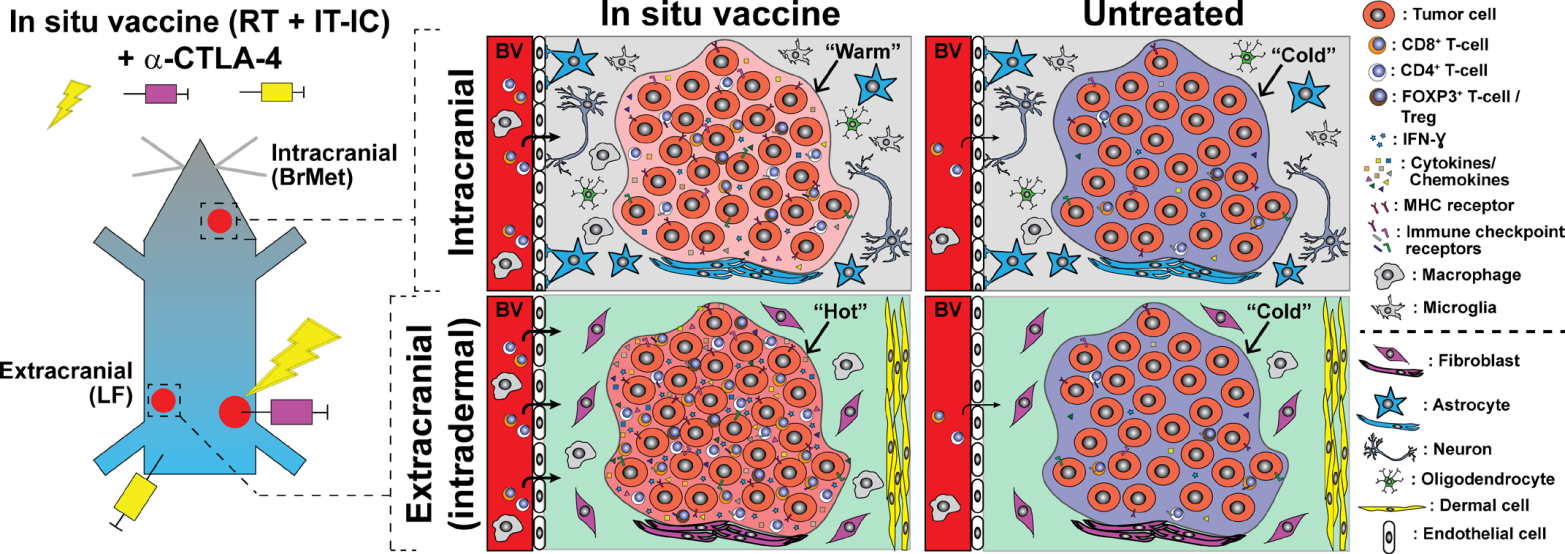
Summary of ISV + α-CTLA-4 regimen antimelanoma effects in extracranial (left flank, LF) compared with intracranial brain metastases (BrMet) for an immunologically “cold” B78 melanoma model. Infiltrating T-cells and immune-related cytokines/chemokines (notably IFN-ɣ) are reduced in BrMet compared with extracranial, but little difference was observed in *Mhc-1* or immune checkpoint inhibitor gene expression. Untreated tumors are indistinguishable in immune microenvironment, but response to ISV + α-CTLA-4 varies dramatically between extracranial and intracranial. Proposed additional factors not investigated here (below dotted line) include tissue-specific cellular environment and differences in T cell penetrance through blood vessels (eg, blood–brain barrier). BrMet, brain met; IC, immunocytokine; IFN-ɣ, interferon-γ; IT, intratumor; RT, reverse transcription.

This effect of tumor location does not appear to be mediated by changes in tumor cell markers of immune susceptibility (eg, *Mhc-1*) or immune checkpoint receptors/ligands (eg, *Pd-l1*). In fact, we see little difference in the expression of these biomarkers at extracranial versus brain melanomas (figure 6), and where observed the differences suggest greater potential for immune checkpoint activation in flank tumors that respond well to ISV. Similar baseline quantification of immune cell infiltrate (figure 6) and baseline inflammatory cytokine/chemokine profiling of untreated flank and brain melanomas were also observed (figure 7). These results are somewhat surprizing because the brain has long been described an ‘immune-privileged’ site.^23^ These findings suggest an absence of intrinsic production of immunosuppressive cytokines or inherent activation of suppressive immune lineages at melanoma tumors in the brain. This is consistent with a model in which there is very little de novo difference between the immune microenvironments of immunologically cold melanoma inside or outside of the brain prior to ISV + α-CTLA-4 treatment (figure 8).

Following ISV + α-CTLA-4, we observed distinct patterns of immune activation and inflammatory response at melanoma sites in the flank and brain. Identified differences in the production of specific cytokines and chemokines in the brain versus flank tumor sites may result from differential activation of immune cells in these locations. Alternatively, at least some of these factors may lead to different antitumor response at these locations. Consequently, such cytokine analyzes could reveal therapeutic targets for future strategies to improve ISV-mediated antitumor response against brain metastases. Our data are consistent with the hypothesis that ISV + α-CTLA-4 activates a systemic antitumor immune response that is detectable at tumors in the flank and in the brain but infiltrating immune cells in the brain tumor microenvironment exhibit reduced activation (figure 8). In support of this, we detect a fivefold difference in the production of the T cell activation marker IFN-ɣ^23 37–39^ in melanoma tumors isolated from brain vs flank of ISV + α-CTLA-4 treated mice (figure 7). Similarly, T cell modulating cytokines such as IL12 (Th1) and IL-4 (Th2)^40^ were detected at low levels in intracranial melanoma but were significantly elevated at extracranial tumor sites. IL-13, a T cell differentiating cytokine,^38^ was also detected at higher levels in flank compared with brain tumors. Notably, this cytokine, along with IL-4, can also affect activity of monocytes, macrophages, dendritic cells and brain-resident microglia.^38^ In contrast, levels of IL-10, an immunosuppressive cytokine,^41^ were not significantly different between these tumor sites (figure 7). Interestingly, we observed that Treg depletion alone does not overcome the inadequate response of brain tumor sites to combined ISV + α-CTLA-4 (online supplementary figure 6). This indicates that depletion of Tregs is not sufficient to overcome immune suppressive mechanisms in the brain and this contrasts with previous observations we have made on the central importance of Tregs in limiting response to our ISV regimen in peripheral tumors.^21^ This does not indicate that Tregs have no role in brain tumor sites but does point to the critical importance of additional suppressive mechanisms.

Interestingly, IHC demonstrates increased myeloid cells and monocytes/macrophages in brain metastases after treatment with ISV + α-CTLA-4 compared with untreated mice, with no change in these cells at extracranial LF tumor with or without treatment (figure 5). Cytokine profiles similarly exhibit differential expression between melanomas from brain and extracranial tumor locations, including a cohort of cytokines known to regulate myeloid cell and monocyte/macrophage recruitment and activity. Specifically, GM-CSF is a strong chemoattractant for myeloid lineages^23 40 42^ and MCP-1, RANTES and MIP-1b modulate macrophage polarization (eg, ‘M1’ proinflammatory vs ‘M2’ immune suppressive states^38^). By recruiting macrophages and maintaining a proinflammatory state, these cytokines may be important in facilitating response to ISV. Many of these cytokines can be produced and released by reactive astrocytes in the brain and can affect microglia function in coordinating and regulating immune response between CNS and periphery.^23 37^ Consequently, in future studies modulation of reactive astrocytes and or microglia may provide a promising approach to better propagating ISV-induced antitumor immunity against brain metastases.^22 37 43–47^

It is intriguing that although the initial ISV response fails to eradicate pre-existing brain melanoma, the robust memory tumor response can eradicate tumor sites in the brain. This may reflect unique aspects of the adaptive memory response versus the immature initial response. Mice exhibiting memory tumor response and depleted of CD4/CD8 T-cells exhibited significantly longer survival than naïve mice (figure 4), supporting a role of non-T-cell immune populations in suppressing brain tumor development such as, but not limited to, phagocytic microglia,^47 48^ non-CNS dendritic or macrophages surveilling within perivascular spaces^40^ or NK cells.^49 50^ Alternatively, this observation may reflect differential tumor growth kinetics, with pre-existing brain tumors at the start of ISV treatment having a ‘head-start’ prior to the delayed development of adaptive immunity following treatment compared with the memory phase. The process of tumor implantation may contribute to this difference, with blood–brain barrier disruption by needle injection enabling immediate immune access to tumor cells in the memory phase, whereas in the treatment phase this perturbation may be healed by the time of adaptive immune response to ISV. The blood–brain barrier is a neurovascular unit that forms a tight barrier and highly regulates molecules entering the CNS.^24^ Other groups have hypothesized that permeation of T cells into brain is a critical hurdle to overcome in propagating antitumor immunity to the brain, with preclinical findings showing that cytotoxic CD8^+^ T cells are primed systemically prior to infiltrating melanoma brain metastases and do not proliferate at brain metastatic sites.^22^ Our data are compatible with this hypothesis. However, it is important to note that a limitation of our model is the injection of tumor cells into brain, which at least temporarily disturbs the blood brain barrier with unknown long-term effects on immune cell infiltration in this area. Notably, since no significant immune cell infiltration is observed along the needle track in the brains of mice receiving control PBS injections (figure 2B), this supports development of tumor-specific immune infiltration of brain melanoma tumors.

Clinical translation of the triple combination treatment approach combining our ISV+ICI regimen is currently underway with a phase I trial currently enrolling patients with advanced melanoma (clinicaltrials.gov #NCT03958383). Close monitoring of patient toxicities and antimelanoma benefit including rates of CNS progression when IC and radiation are added to ICIs are critical endpoints in this study. The current preclinical data supports excluding patients with known and untreated brain metastases from this treatment combination, but suggests a benefit could be observed in reducing future progression of disease in the brain among responding patients.

## Conclusions

Overall, our results demonstrate that ICI combined with an RT +IT IC ISV regimen elicits immune responses against melanoma tumors in the brain. This suggests potential for development of highly efficient immunotherapy treatments for melanoma brain metastases; however, further enhancement of this response or additional combinatorial treatments will be required—especially for effective treatment of pre-existing brain metastases. The interaction between adaptive antitumor immunity and the brain microenvironment are not completely elucidated.^23 51 52^ Our preclinical model is well suited for the future studies that investigate these specific mechanisms. It is clear from this work and from other preclinical and clinical studies that antitumor T cells can elicit response at intracranial tumor sites (figure 1).^1–3 22^ Additional studies suggest potential effector and/or immunomodulatory roles for brain-resident microglia^47^ and astrocytes.^43 44^ These data suggest possible ways to enhance immunotherapies against brain metastases by further elucidating the unique effects of the brain microenvironment on adaptive antitumor immunity.
