# Supplementary material for: In situ vaccination at a peripheral tumor site augments response against melanoma brain metastases
Source: J Immunother Cancer. 2020 Jul 19;8(2):e000809. doi: 10.1136/jitc-2020-000809 (PMC7371368; doi:10.1136/jitc-2020-000809)
Supplement: Supplementary data [file jitc-2020-000809supp001.pdf]

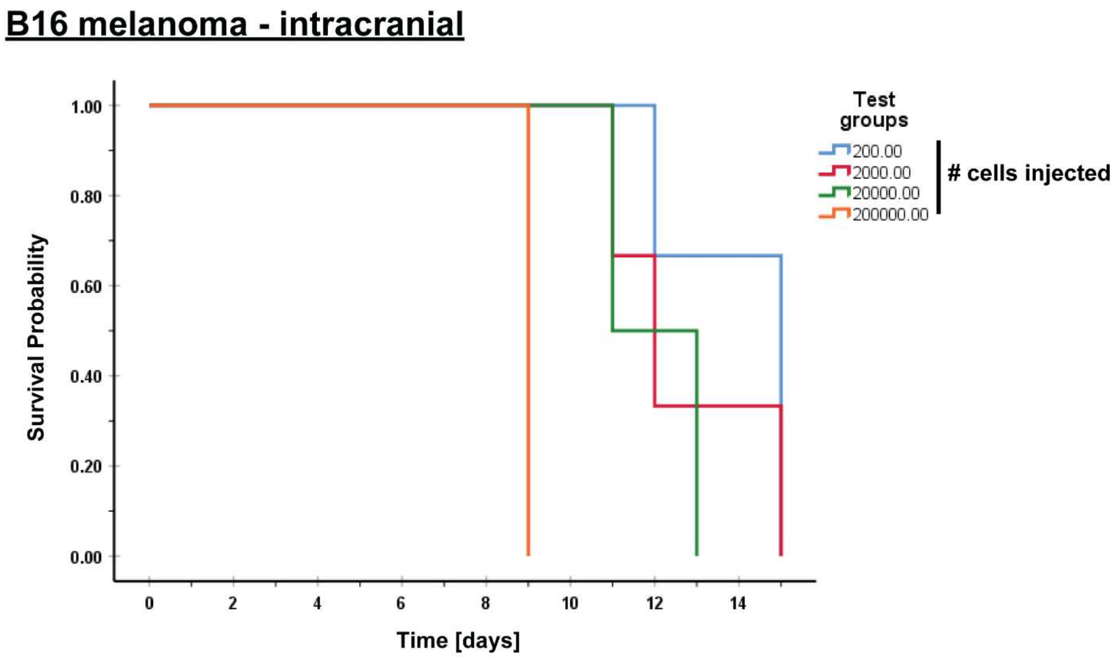

Supplementary Figure 1: Survival time for mice intracranially injected with B16 (GD2$^-$) melanoma cells ranging from 200 – 200,000 cells.

Clark PA, *et al. J Immunother Cancer* 2020; 8:e000809. doi: 10.1136/jitc-2020-000809

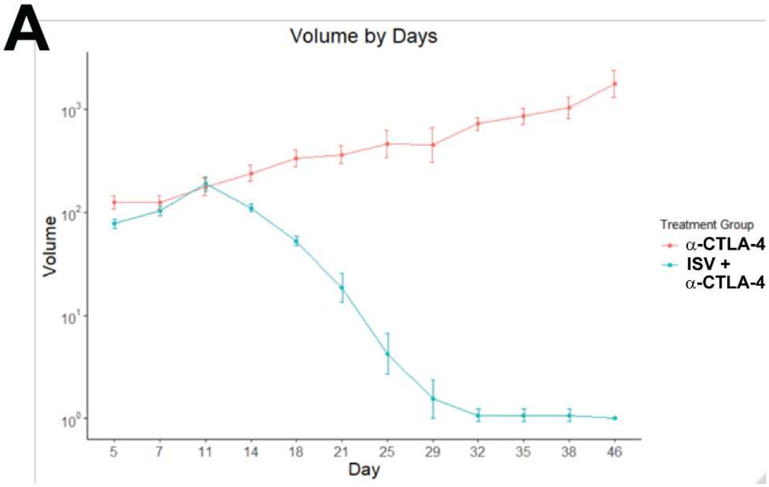

B

Day	Treatment Group	n	mean_vol	sd_vol	p_value
5	A	8	123.99	11.92	<0.001
5	B	22	76.08	8.29	
7	A	8	123.75	11.49	0.07
7	B	22	102.71	12.53	
11	A	8	174.38	21.82	0.61
11	B	22	186.77	30.45	
14	A	8	237.90	25.58	<0.001
14	B	22	109.18	10.54	
18	A	8	332.56	39.67	<0.001
18	B	22	51.70	5.60	
21	A	8	360.41	45.10	<0.001
21	B	22	17.36	5.80	
25	A	8	456.20	85.36	<0.001
25	B	22	3.24	1.52	
29	A	8	445.62	107.36	<0.001
29	B	22	0.55	0.24	
32	A	8	715.28	62.96	<0.001
32	B	22	0.08	0.01	
35	A	8	848.70	92.72	<0.001
35	B	22	0.08	0.01	
38	A	8	1022.44	152.44	<0.001
38	B	22	0.08	0.01	
46	A	8	1752.89	334.04	<0.001
46	B	22	0.00	0.00	

Supplementary Figure 2: (**A**) Log10-transformed tumor growth curve of Fig. 1B. (**B**) Log10 transformed data was used to determine significantly different tumor growth rates at multiple time points. Treatment groups, A: α-CTLA-4 only, B: ISV + α-CTLA-4.

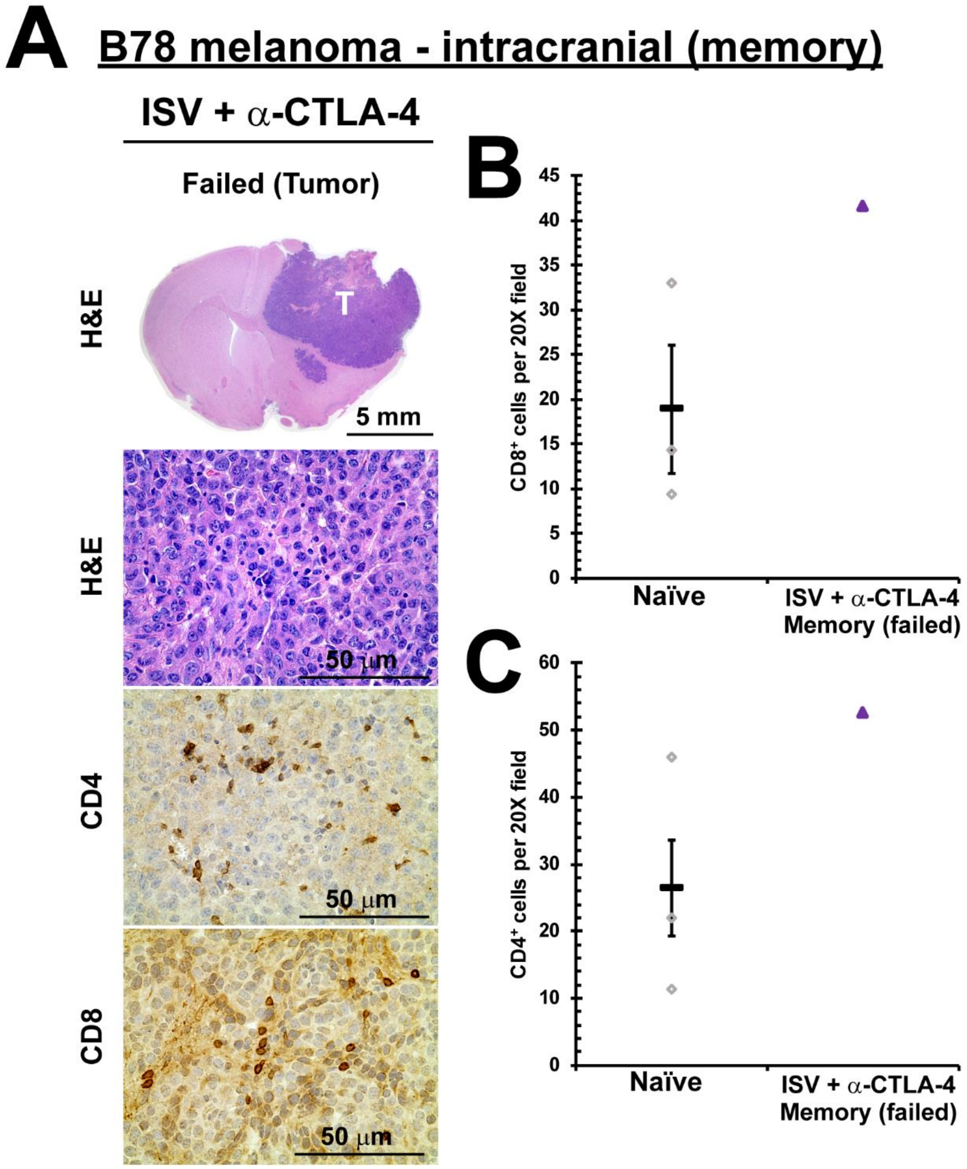

Supplementary Figure 3: Immune cell analysis of 1 (of 12) mouse that failed to establish immune memory in CNS. (**A**) Immunohistochemistry representative images of $CD4^+$ and $CD8^+$ within developed brain tumor (T: tumor, brown: positive immunolabeling, H&E: hematoxylin and eosin). Quantification of $CD8^+$ (**B**) and $CD4^+$ (**C**) T-cells demonstrated increases in brain tumor of 1 (of 12) mouse that failed to establish immune memory compared to naïve mice (euthanized due to brain tumor burden, not time-matched).

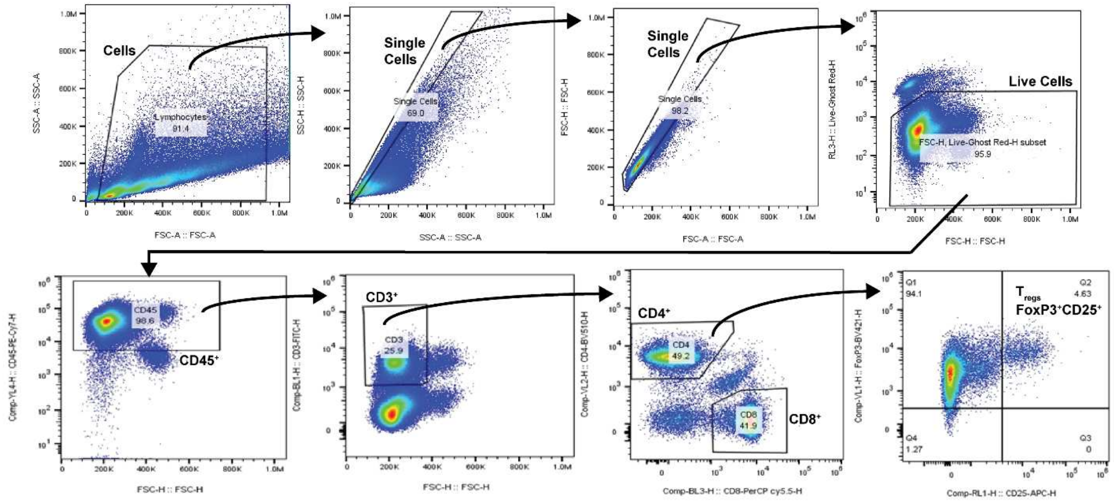

Supplementary Figure 4: Flow cytometry gating strategy.

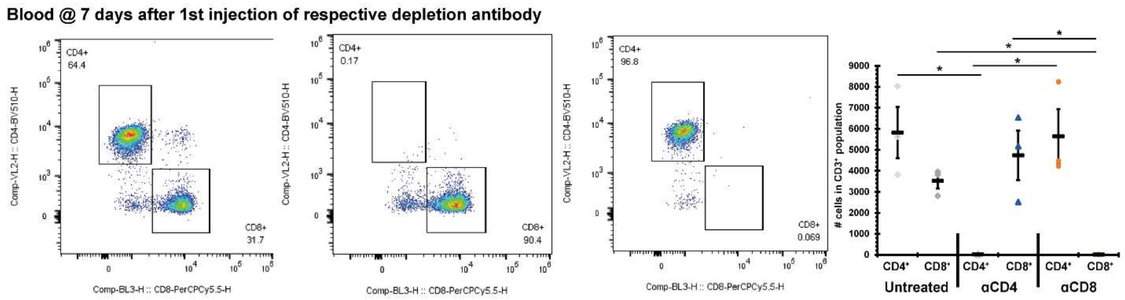

Supplementary Figure 5: Depletion efficiency for α-CD4 and α-CD8 antibodies. Injection (i.p.) of α-CD4 and α-CD8 antibodies resulted in significant >97% depletion of $CD4^+$ and $CD8^+$ cells, respectively, in mouse blood 7 days after first administration (*$p<0.05$, mean±S.E., n=3 mice in a single animal experiment, ANOVA with post-hoc Bonferroni).

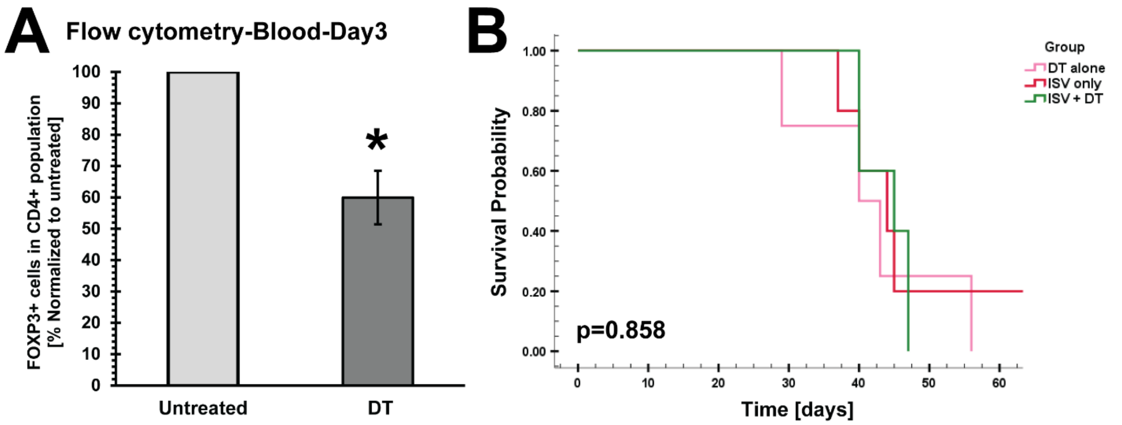

Supplementary Figure 6: FOXP3$^+$ cell depletion in DEREG mouse model. (**A**) Flow cytometric analysis at day 3 post diphtheria toxin (DT) injection (*p<0.05; mean±S.E., n=3 mice total in two separate animal experiments, Student's *t*-test). (**B**) Survival curve for mice harboring intracranial and extracranial B78 tumors, and receiving ISV and/or DT treatment (p=0.858, Kaplan-Meier, n≥4 in a single animal experiment).
